# Synthesis and Structural Characterization of Sequential Structure and Crystallization Properties for Hydrophilic Modified Polyester

**DOI:** 10.3390/polym12081733

**Published:** 2020-08-03

**Authors:** Lina Sun, Liqian Huang, Xueli Wang, Hongmei Hu, Juanzi Guo, Ruishu Zhu, Shuang He

**Affiliations:** 1 Key Laboratory of Textile Science & Technology of Ministry of Education, College of Textiles, Donghua University, Shanghai 201620, PR China; 1185060@mail.dhu.edu.cn (L.S.); 1189131@mail.dhu.edu.cn (H.H.); 2190200@mail.dhu.edu.cn (J.G.); 2180047@mail.dhu.edu.cn (R.Z.); 2190069@mail.dhu.edu.cn (S.H.); 2 Textile Technology Innovation Center, Donghua University, Shanghai 201620, China

**Keywords:** copolyester, hydrophilic, sequential structure, crystallization properties

## Abstract

The hydrophilic copolyester polyethylene terephthalate (PET) (ENCDP-X) was successfully synthesized by chemical modification consisting of copolymerization and blending and the comonomers, including sodium isophthalate-5-sulfonate (SIPE), polyethylene glycol (PEG), 2,2-dimethyl-1,3-propanediol (NPG) and matting agent TiO_2_ with different content. Moreover, the structural characterization of sequential structure, crystallization and thermal properties were studied. The results showed that the comonomers were successfully embedded in the copolyester, the actual molar ratio in the copolyester was consistent with the relative feed ratio and the degree of randomness was calculated to be 0.99, showing that the random copolymers synthesized during the melt polycondensation process and the chemical structure was roughly consistent with the expected molecular chain sequence structure. The thermal parameters of the modified copolyester, containing the glass transition temperature (*T*_g_), melting point (*T*_m_), crystallinity (*X*_c_) and thermal degradation temperature, were decreased, and the cold crystallization temperature (*T*_c_) was increased. In addition, with the increasing of the TiO_2_ content, it improves the thermal performance of the copolyester and it is beneficial to processing and application. The above conclusion is further verified by non-isothermal kinetic analysis. In addition, the copolyester exhibited the better hydrophilicity than pure PET.

## 1. Introduction

Polyethylene terephthalate (PET), which is known for its excellent mechanical properties and chemical resistance, consisting of high tenacity, excellent dimensional stability and good heat resistance, have been widely commercialized for many years in the textile industry [1,2,3]. However, its hygroscopicity is poor and the fiber moisture regain is only about 0.4%, due to its high crystallinity, compact amorphous structure and shorting of active hydrophilic groups [4,5]. In addition, owing to the shortage of cotton resource and the excess capacity of PET fiber industry, the hydrophilic polyester fibers have become the research trend of modified polyester in the textile field [6,7,8,9].

Many studies and practices have proved that introduction of additional monomer into PET molecular chain changes the hydrophilic properties of PET fibers potentially by introducing hydrophilic groups [10,11] or reducing the molecular regularity and crystallinity of PET [5,12]. Wang, Qiong et al., [13] used terephthalic acid (PTA), ethylene glycol (EG) and dihydroxyethyl isophthalate-5-sulfonate (SIPE) as the third monomer, and the different content polyols as the fourth monomer, synthesizing a series of hydrophilic copolyester, and the fiber moisture regain was generally only 0.6–0.8%. Wang Huaping [14] used polyhydric alcohols, diols and polyethylene glycol to prepare hydrophilic polyester fibers by utilizing the polyhydroxy active sites and the ether bond in the PEG segment to improve hygroscopic properties of the fibers. However, the polymerization is prone to crosslinking reactions. In the current research, the improvement of the hygroscopicity for copolyester PET fibers is still limited [15], and the moisture regain is generally less than 1% [16].

In our previous research, it was showed that the incorporation of monomer dimethyl-1,3-propylene glycol (MPD/NPG) in the polymerization process led to less perfect crystals and reducing the crystallinity, improving the hydrophilic properties of the copolyester [4,17]. In this work, the hydrophilic copolyester was designed and synthesized by introducing additional monomers into the PET molecular chain to improve the hydrophilic properties of the fibers, combining chemical modification methods of copolymerization and blending by incorporating 2,2-dimethyl-1,3-propanediol (NPG) as a branched modified monomer to further reduce the crystallinity and increase the binding capacity with water, and blending TiO_2_ to provide access with water and further improve the hydrophilicity, based on diethylene isophthalate-5-sulfonate (SIPE) and polyethylene glycol (PEG-2000), increasing from the more aspects hygroscopicity of modified polyester.

However, when different monomers are added to the copolymerization system, the copolymerization process of various monomers is difficult to control and it is difficult to ensure the quality of the copolymer. In addition, the properties of the copolymer depend not only on the composition of the comonomer, but also on the sequence distribution and randomness of the constituent comonomers [18,19,20]. The latter directly restricts the macromolecular configuration and conformation [21,22], which in turn affects the crystallization process of macromolecular and the performance of products. Therefore, exploring the sequential structure of resulted copolymer is highly necessary, including that the comonomer sequence distribution is characterized by nuclear magnetic resonance (NMR) spectroscopy and the ideal microscopic sequence structure is analyzed by a statistical model [23,24]. To date, the characterization of binary copolymers has been widely reported [25,26,27]. However, the research on the sequence distribution for third or more comonomers of copolyester is rare.

The paper focused on the characterization of the structure and properties for synthetic copolymer. The effects of modified monomers on sequential structures, crystalline structures, thermal properties and hydrophilicity of prepared copolyester were mutually compared and elucidated in detail by ubbelohde viscometer, IR-raman spectrometer, nuclear magnetic resonance (^1^H- and ^13^C-NMR), two-dimensional nuclear magnetic resonance (^1^H-^13^CCOSY), differential scanning calorimetry (DSC), thermogravimetric analyzer (TGA) and contact angle meter of OCA15EC (China).

## 2. Experimental

### 2.1. Materials

The purified terephthalic acid (PTA) was supplied by Hengli Chemical, Suzhou, China; ethylene glycol (EG) was supplied by Yangzi Petrochemical, Nanjing, China; sodium-5-sulfo-bis-(hydroxyethyl)-isophthalate (SIPE) was supplied by Wujiang Wanda, Suzhou, China; Poly (ethylene glycol) (PEG-2000) and 2,2-dimethyl-1,3-propanediol (NPG) was supplied by Sinopharm Chemical, Shanghai, China; Titanium Dioxide (TiO_2_) was supplied by Jianghuai Chemical, Hefei, China; antimony trioxide (C_6_H_12_O_6_Sb_2_), trimethyl phosphate (C_3_H_9_O_4_P) and sodium acetate (CH_3_COONa) was supplied by Sinopharm Chemical, Shanghai, China. These were not purified further.

### 2.2. Synthesis of Copolyester

**Scheme 1:**
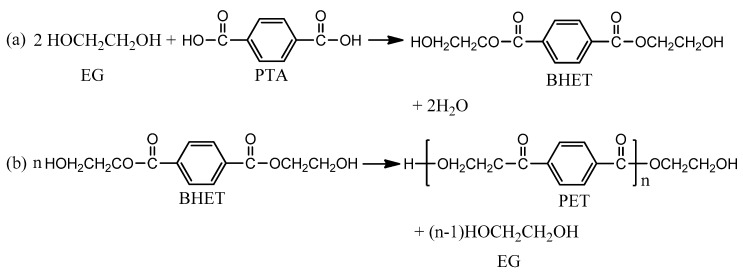
Synthesis of (**a**) ethylene terephthalate (BHET) and (**b**) of the macromer polyethylene terephthalate (PET) di-hydroxy ended.

Ordinary PET was synthesized by direct esterification in a 5L reactor. PTA and EG were fed into the reaction with molar ratio of 1:1.2, and then antimony trioxide (0.05 wt %) and trimethyl phosphate (0.07 wt %) were sequentially incorporated into esterification reaction. Esterification was carried out at 240 °C for 2–3 h under nitrogen atmosphere to prepare polyester ethylene terephthalate (BHET). At the end of esterification reaction, the matting agent TiO_2_ was incorporated into the reactor. Subsequently, polycondensation reaction occurred at 260–270 °C for 2.5–3.5 h and the pressure of 0.3 MPa was continually decreased to 100 Pa. Then chips of polyesters were discharged, granulated and dried to prepare ordinary PET with different TiO_2_ content. Furthermore, the synthetic route is as Scheme 1.

**Scheme 2:**
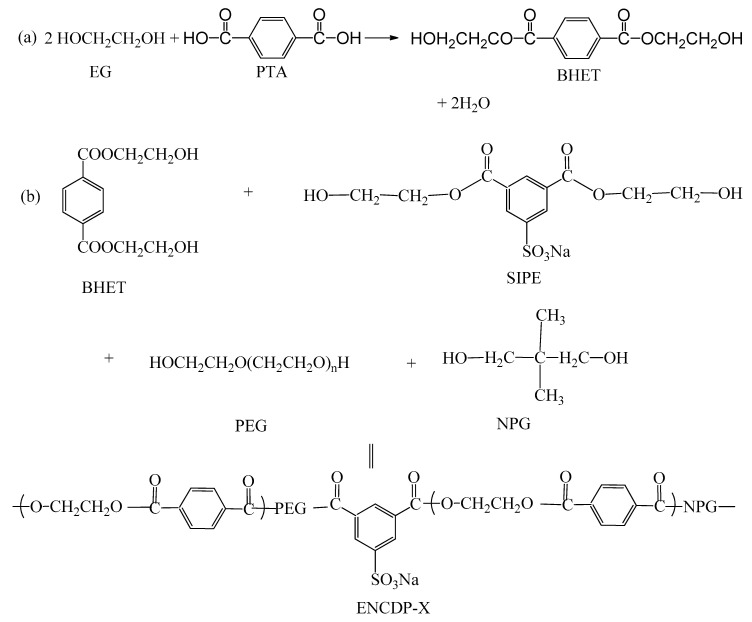
Synthesis of (**a**) ethylene terephthalate (BHET) and (**b**) the modified copolyester polyethylene terephthalate (ENCDP-X).

For the preparation of hydrophilic copolyester, after the first esterification reaction, the modified monomers were added into the second esterification reactor, and the monomer SIPE (molar ratio of SIPE to PTA= 1.75/100) and sodium acetate (0.005 wt %, preventing the ionization for SIPE) were added into the reactor at 210 °C for 20 min. Then, PEG (molar ratio of PEG to PTA = 0.8/100), NPG (molar ratio of NPG to PTA = 4.3/100) and matting agent titanium TiO_2_ were added into the reactor together at 230 °C for 1.5h. Subsequently, the polycondensation reaction was carried out to prepare hydrophilic copolyester ENCDP-X with different TiO_2_ content for experiment. All samples were numbered as shown in Table 1 and the synthetic route is as Scheme 2.

The synthetic routes are schematized in Scheme 1 and Scheme 2.

### 2.3. Characterization of Copolyester 

**Infrared spectroscopy analysis.** It was measured by NEXUS-670 infrared-raman spectrometer (Nicollet, Minnesota, US). The scanning range is 4500–400 cm^−1^, the resolution is 2 cm^−1^ and the samples were scanned 10 times with the attenuated total reflection ATR method.

**Nuclear Magnetic Resonance analysis.**^1^H-NMR, ^13^C-NMR and ^1^H-^13^CCOSY were carried out on the nuclear magnetic resonance spectrometer (Avance 400 Bruker, Billerica, MA, USA). Samples of around 5 mg were put into special sample tube and completely dissolved in deuterated reagent of trifluoroacetic acid (TFA) with the test frequency of 400 MHz. All chemical shifts are reported in ppm and tetramethylsilane (TMS) was used as the internal standard.

**Differential Scanning Calorimetry (DSC) analysis.** The thermal properties of the copolyester were carried out on the TA Instrument DSC-Q20 Differential Scanning Calorimeter (PerkinElmer, Waltham, MA, USA). The test was carried out under nitrogen atmosphere and the flow rate of the nitrogen gas stream was 50 mL/min. The 7mg sample was put into an aluminum crucible and sealed. The samples were melted at 280 °C with the heating rate of 20 °C /min, and then holding the temperature at 280 °C for 3 min to remove thermal history. Subsequently, the temperature was rapidly cooling to 0 °C with the cooling rate of 50 °C/min, holding the amorphous state and exploring the ability of cold-crystallization. Thereafter, the samples were reheated to 280 °C at the heating rate of 10 °C/min and then holding the temperature at 280 °C for 3 min. Then cooling to 0 °C at the cooling rate of 10 °C/ min. Which were characterized the thermal properties of the samples.

**Thermogravimetric analysis (TGA).** It was measured by TGA-4000 Thermogravimetric Analyzer (PerkinElmer, Waltham, MA, USA) under nitrogen atmosphere. 6 mg samples of the copolyester were gradually heated from 25 to 600 °C at the heating rate of 10 °C/min, and the residues were naturally cooled to room temperature.

**The intrinsic viscosity (IV) analysis.** According to GB/T 14190-2008 test standard, the intrinsic viscosity of the samples was tested by NYC-2 semi-automatic ubbelohde viscometer (Silda Scientific Instruments Co., Ltd., Shanghai, China). Copolyester chips were dissolved in 25 mL mixed solutions of phenol/tetrachloroethane (1:1, *w*/*w*), the tested temperature was 25 °C and repeated three times. wherein the viscosity average molecular weight (*M*_η_) was calculated by the Mark–Houwink equation, (2), listed in Table 2.

(1) $$\eta =KM_{\eta }{}^{\alpha }$$

(2) $$\ln\left[\eta \right]=\ln K+\alpha \ln M_{\eta }$$

where *K* is equal to 2.1 × 10^−4^ and *α* is equal to 0.8.

The molecular weight has a significant impact on the mechanical strength and thermal resistance for its products. Owing to the lower molecular weight, it will be difficult for polymer molding and without mechanical strength. However, the higher molecular weight will increase the flow viscosity of the polymer and causing processing difficulties. Therefore, the molecular weight of the polymer should be controlled within a certain range, taking into account the requirements of both application and processing.

The results of intrinsic viscosity (IV) and viscosity average molecular weight (*Mη*) of copolyester are listed in Table 2 shows that *Mη* of all copolyester from 15,000 to 19,000 g/mol, which are satisfied requirements for spinning.

**Contact angle analysis.** It was measured by the optical contact anglemeter of OCA15EC (Beijing, China). The copolyester chips were melted at 260 °C for 2 min, then pressed into pieces by plate vulcanizing machine and cooled downing quickly by frosty iron nuggets. Measured five times and averaged.

## 3. Results and Discussion

### 3.1. Fourier Infrared Spectroscopy

**Figure 1:**
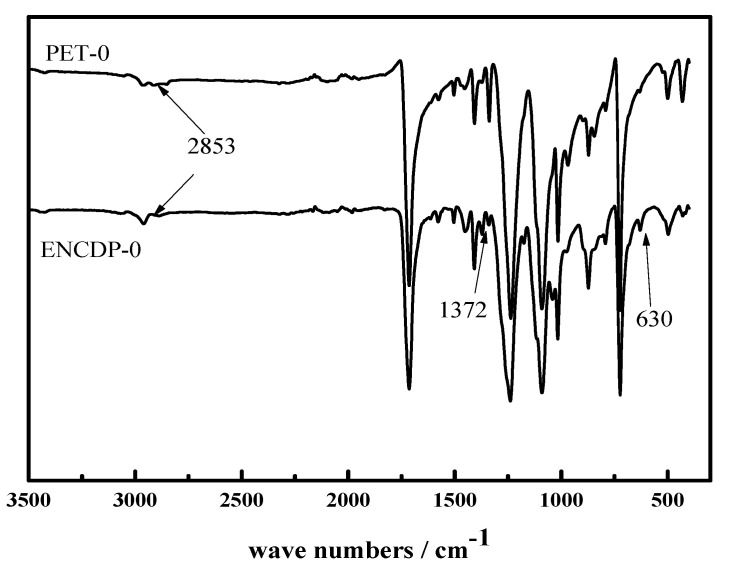
FTIR spectra of PET-0 and ENCDP-0.

The infrared spectroscopy analysis is performed to determine the composition of the copolymers. It is inferred whether the comonomers are incorporated into the molecular chain according to the position and shape of the absorption peak in the spectrum, apart from the matting agent TiO_2_ can be directly judged by visual sense. The chemical spectra of sample PET-0 is same as to PET-1/ PET-2, and the chemical spectra of sample ENCDP-0 is same as to ENCDP-1/ ENCDP-2. Selecting PET-0 and ENCDP-0 as the targets, and the others will not be repeated. The spectral patterns of PET-0 and ENCDP-0 are shown in Figure 1, and the wave numbers and vibration mode of chemical groups of copolymers are shown in Table 3.

In Figure 1, it is revealed clearly that there are three distinct characteristic peaks between PET-0 and ENCDP-0 at 630, 1372 and 2853 cm^−1^. The absorption peak at 630 cm^−1^ is caused by S–C stretching vibration, indicating that the SIPE monomer is successfully incorporated into the PET macromolecular chain. The copolyester ENCDP-0 has an absorption peak at 1372 cm^−1^, which is attributed to the symmetric deformation vibration of the methyl group C–H in the NPG monomer, indicating that the NPG monomer is successfully incorporated into the macromolecule. In addition, copolyester ENCDP-0 owns weak stretching vibration peaks at 2853 cm^−1^ of –CH_2_– in PEG monomer, indicating that the PEG monomer successfully incorporated into the macromolecule. It was confirmed by infrared spectroscopy that all the modified monomers have been successfully incorporated into the copolymer macromolecule.

### 3.2. Nuclear Magnetic Resonance (NMR) Analysis

More chemical structure details of copolyester were further characterized by NMR spectroscopy. The chemical groups will be ascertained basing on the chemical shifts in the ^1^H-NMR spectra and the relative molar ratio of H protons could be obtained by calculating the areas of resonance peaks [28,29]. ^13^C-NMR characterizes the macromolecular sequence, based on the position and content of the splitting peak of quaternary carbon. ^1^H-^13^CCOSY can be observed the coupling signals of the hydrogen spectrum and the carbon spectrum, and concluded the connection of the copolymerized monomers. The ^1^H-NMR and ^13^C-NMR spectra of ENCDP-0 copolymers are consistent with ENCDP-1/ENCDP-2, and which will not be repeated. As an example, the ^1^H-NMR and ^13^C-NMR spectra of ENCDP-0 copolymer are indicated.

### 3.3. ^1^H-NMR Analysis

**Figure 2:**
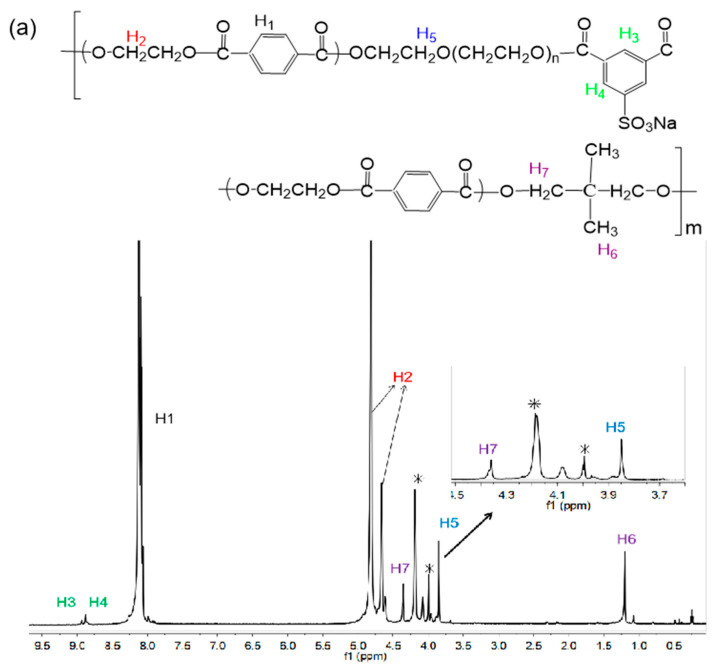

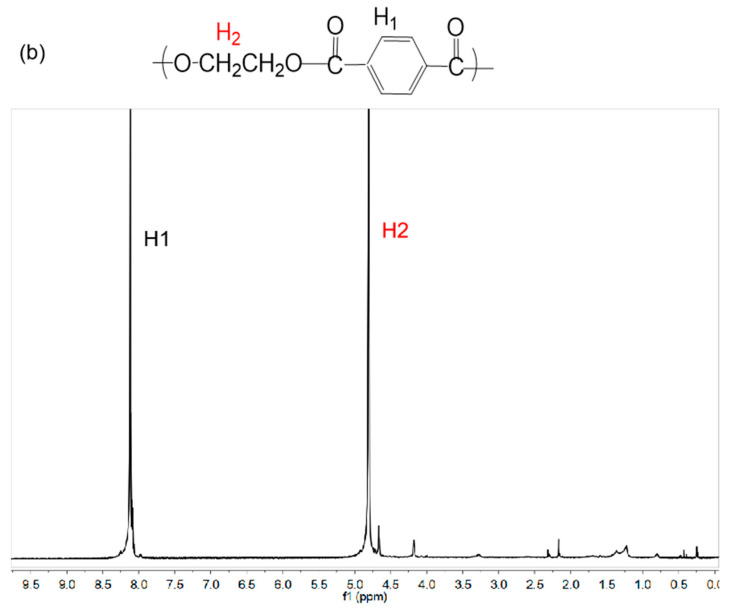
^1^H-NMR spectra of (**a**) ENCDP-0 and (**b**) PET-0.

Figure 2 shows the ^1^H-NMR spectra of ENCDP-0 and PET-0 samples, which revealed that the chemical shifts of ENCDP-0 are distinctly different from PET-0, causing by H protons of PTA, EG, SIPE, and PEG units. Specifically, the hydrogen protons H1 are derived from aromatic protons in the PTA unit, and the corresponding chemical shifts are around 8.20 ppm. The aliphatic hydrogen proton H2 are derived from the EG unit, and the corresponding chemical shift is about 4.7 ppm. The aromatic hydrogen protons H3 and H4 are derived from the third monomer SIPE unit, and the corresponding chemical shifts are around 8.70 and 8.90 ppm. The aliphatic hydrogen proton H5 are derived from the fourth monomer PEG2000 unit, and the corresponding chemical shift are around 3.9 ppm. The methyl and methylene protons of H6 and H7 is derived from the fifth monomer NPG unit, and the corresponding chemical shifts are around 1.20 and 4.4 ppm. The aliphatic hydrogen proton Hx (✳) are mainly derived from the unit of the by-product diethylene glycol (DEG), which are mainly caused by the chemical hydrolysis reaction, and corresponding chemical shift are around 4.0–4.2 ppm.

According to the relative area of the integral curve of each resonance peaks in the ^1^H-NMR spectrum, the relative molar ratio of the modified monomer in the copolyester is quantitatively calculated by the following equation. The equation which is used to calculate the data is summarized in Table 4.

(3) $$\frac{n_{SIPE}}{n_{PTA}}=\frac{\frac{1}{3}\left(H_{3}+H_{4}\right)}{\frac{1}{4}H_{1}}$$

(4) $$\frac{n_{PEG2000}}{n_{PTA}}=\frac{\frac{1}{45}\times \frac{1}{4}H_{5}}{\frac{1}{4}H_{1}}=\frac{H_{5}}{45H_{1}}$$

(5) $$\frac{n_{NPG}}{n_{PTA}}=\frac{\frac{1}{6}H_{6}}{\frac{1}{4}H_{1}}=\frac{2H_{6}}{3H_{1}}\;\mathrm{or}\;\frac{n_{NPG}}{n_{PTA}}=\frac{\frac{1}{4}H_{7}}{\frac{1}{4}H_{1}}=\frac{H_{7}}{H_{1}}$$

It can be seen from Table 4 that the actual composition of the modified monomers in the copolyesters is close to the corresponding feed amount, which indicates that most of the modified monomers have been incorporated into the polymerization and successfully incorporated into the macromolecule.

### 3.4. 2D NMR HETCOR Spectra Analysis

**Figure 3:**
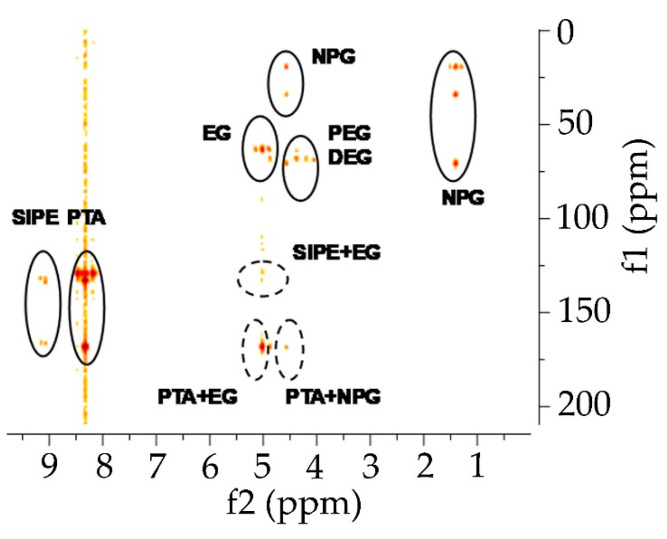
The ^1^H-^13^C heteronuclear shift correlation spectrum (HETCOR) of ENCDP-0.

The Heteronuclear chemical shift correlation Spectroscopy(HETCOR) characterizes the relationship between the ^13^C nucleus and ^1^H protons. The f1 and f2 axes usually represent different nuclei, the carbon spectrum is on the right, the hydrogen spectrum is on the bottom and the corresponding correlation spectrum are found on the opposite side of each axis. Their correlation peaks indicate ^13^C and ^1^H originated from 2–4 chemical bonds, and the coupling relationship is represented by ^n^J_CH_. The correlation mode of the modified monomer in the copolyester is represented by the HMBC spectrum, which are shown in Figure 3.

The vertical line is drawn from the f2 axis (C atom) and the f1 axis (H protons), and the intersecting peaks of the two vertical lines directly reflect the coupled signals of the two atoms. It can be seen from the Figure 3 that the solid circle is the itself coupling of C and H of the comonomer. The absence of the agglomeration signal of the modified monomer is due to the low content of the modified monomer and the feeding process. In order to avoid agglomeration, they are first dissolved in ethylene glycol, uniformly stirred, and then slowly added during the feeding process. The dashed circle is the coupling signal between the C and the H of different comonomers. There are intersecting signals between PTA and EG, SIPE and EG, and PTA and NPG, and there is no signal between PTA and SIPE, as well as SIPE and NPG. In addition, the chemical shifts of the H protons of the PEG and the byproduct DEG are well-matched and cannot be distinguished, so the description of the PEG-related sequence structure is not considered. In short, signal assignment was accomplished by comparing the spectra obtained from different copolymer compositions and it was supported by relevant data provided by 2D NMR HETCOR spectra.

### 3.5. ^13^C-NMR Analysis

The properties of the copolymer depend not only on the comonomers that make up the macromolecular chain, but also on the sequence distribution. The sequence distribution includes randomness, alternation and block copolymerization, which are judged by the value of the randomness. The most powerful method for comonomer sequence distribution is NMR spectroscopy, and a statistical model is usually used to summarize the ideal microscopic sequence structure. Comparison of theoretical and experimental data provides an estimate of the randomness. The number of resonances that can be observed by the number of dyads, triads and higher sequences in which the two monomers may be arranged^24^. For example, if F_B_ and F_S_ represent the composition ratio of isophthalic and terephthalate in the copolymer, and according to the the Bernoulli distribution model, XB^2^, 2XBXS and XS^2^ can, respectively, represent the theoretical content of the BB, BS and SS dyads sequences in the copolyester. Similarly, the composition ratio of the triads sequence can also be calculated in the similar method, and the sequence structure of copolyester synthesized in our experiment are investigated on the basis of the sequence distribution, randomness and sequence length.

The subject adopts five-units copolymerization, and the characterization of molecular sequences by NMR spectroscopy is difficult to achieve. According to the actual process of polymerization, PTA and EG of polymerized monomers are regarded as a substance BHET. Moreover, the chemical shift of the hydrogen proton for the modified monomer PEG and the by-product DEG is highly coincident, and the sequence distribution of the fourth monomer is not considered at present. Therefore, the five-unit copolymerization will be simplified to triads copolymerization for characterization. The substance BHET is abbreviated as B, the monomer SIPE is abbreviated as S, and the monomer NPG is abbreviated as N. The entire molecular chain structure is centered on B, which is briefly described as: BBB, BBS, SBS, BBN, NBN, SBN. However, since the composition ratio of the monomer S is litter, the ^13^C NMR spectrum does not show the characteristic peak of SBS.

**Figure 4:**
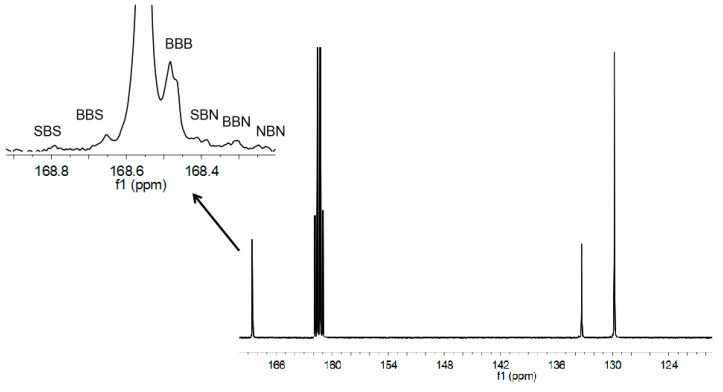
^13^C-NMR spectra of ENCDP-0.

Figure 4 shows the chemical shifts of the carbon atom and the splitting peaks of the carbon of carbonyl groups belonging to the aliphatic-aromatic ester units, and calculates the average sequence lengths L_S_ and L_N_ of the modified monomer links according to the integrated area of the characteristic peaks. The degree of sequence randomness of ENCDP-0 can be calculated by the following equations.

L_B_=(F_BBB_+F_BBS_/2+F_SBS_+F_BBN_/2+F_NBN_)/(F_BBS_/2+F_SBS_+F_BBN_+F_NBN)_ (6)

Ls=(F_BBS_/2+F_SBS_+F_SSS_)/(F_BBS_/2) (7)

L_N_=(F_BBN_/2+F_NBN_+F_NNN)_/(F_BBN_/2) (8)

where Fi denotes the mol fraction of each sequence.

The calculation equations of the randomness B_i_:

Bs=1/L_B_+1/Ls (9)

B_N_=1/L_B_+1/L_N_ (10)

For the random copolyester, the degree of randomness B_i_ should be equal to 1. For the alternating copolyester, the degree of randomness B_i_ is equal to 2. If B_i_ is below 1, the repeat-units are supposed to be arranged in blocks.

For sample ENCDP-0, F_B_, F_S_ and F_N_ are 95.07%, 1.64% and 3.29%, respectively, according to ^1^H-NMR data. The molar fraction of the dyads sequence distribution is calculated by the Bernoulli distribution theory. For the binary system of B and S, F_BB_, F_BS_ and F_SS_, this is 95.88%, 3.12% and 0.03%, respectively; for the binary system of B and N, FBB, FBN and FNN, this is 94.03%, 6.26% and 0.11%, respectively. The theoretical molar ratio of triads sequence distribution is calculated by a similar method, and the actual molar ratios were obtained by the integral area of the ^13^C-NMR characteristic peak. The experimental and theoretical values for the different sequence structure of the copolymer macromolecules are shown in Table 5:

It can be showed from the above table that the experimental values of the sequence structure of the ENCDP-0 are substantially consistent with the theoretical values, which confirm that the triad sequence distribution of the copolymer follows the Bernoulli distribution model.

Table 6 shows the experimental average sequence lengths and randomness of copolyester are consistent with the ideal polycondensation statistics with randomness. The randomness Bi is very close to 1, which is a highly expected result since the occurrence of transesterification reactions tends to be random copolyester in the molten phase.

### 3.6. DSC Analysis

As can be seen from Table 7, there is only one glass transition in DSC curves, implying that no second phase exists in the copolyester ENCDP-X and the glass-transition temperature (*T_g_*) of ENCDP-X decreases with the adding of modified monomers. This indicates that the incorporation of the methyl pendant groups (–CH_3_) from NPG units disrupted the regularity of the molecular chain and increased the free volume and amorphous regions, leading to less energy required for chain molecular motion and rearrangement [16]. However, *T*_g_ increases gradually with the increasing content of TiO_2_, which is caused by the TiO_2_ enhance the crystallinity of the polyesters and form the perfect crystalline state, leading to more energy required for rigid chain molecular motion. In addition, the effects of adsorption and steric hindrance from TiO_2_, in turn, hinder the movement of macromolecules and causing the *T*_g_ increase. The *T*_g_ of samples (PET-1 and PET-2) is gradually increased with the increase of TiO_2_ content, which reason is the same as the above copolyester.

**Figure 5:**
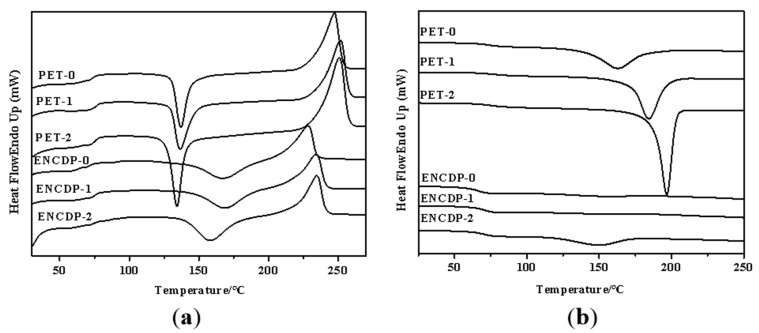
(**a**) Curves of copolymers; (**b**) cooling curves of copolymers.

It can be seen from Figure 5 that, comparing to ordinary polyester PET-0, the crystallization ability of the samples ENCDP-X is weakened. The main reasons are that the methyl groups the modified monomer NPG disrupt the regularity molecules and the strong polarity of the sulfonic acid group on the SIPE benzene strengthen the steric hindrance, causing the cold-crystallization temperature increase, and the crystallization temperature, melting temperature decrease [30]. With the increase of the TiO_2_ content in the samples ENCDP-X, TiO_2_ increase the crystallization ability and form perfect grains, causing the cold-crystallization temperature reduces, the crystallization temperature and melting temperature increases, which plays a promoting crystallization role. In summary, TiO_2_ achieves the effect of matting agent, but the crystallization effect is still not significant, which achieves our purpose of designing macromolecular structure. However, TiO_2_ plays a significant crystal nuclei role for samples (PET-1 and PET-2).

In summary, when both the modified monomer and TiO_2_ in the copolyester are incorporated, the influence of the modified monomer is dominant. That is, when the copolyester has a stronger crystallization ability, the crystal nuclei effect of TiO_2_ is more significant. However, the incorporating of TiO_2_ not only improves the thermal performance of the copolyester which is beneficial to processing and application, but also provides more channels for moisture enter the copolyester to improve hygroscopicity, and which can meet the design requirements.

In order to further study the effect of modified monomers and TiO_2_ on the crystallization behavior of copolyester, the non-isothermal crystallization kinetics of samples were explored by DSC. Moreover, the process of non-isothermal crystallization will be closer to the actual spinning process. The Avrami equation is directly used to analyze the cold-crystallization processes on the DSC curves of the six samples, and then the crystallization kinetic parameters are studied and summarized based on the Jeziorny method.

The relative crystallinity *X_t_* of the samples at different times can be calculated by Equation (11):

(11) $$X_{t}=\frac{\int^{\;}\frac{T}{T_{0}}\left(\frac{dH_{T}}{dT}\right)dT}{\int^{\;}\frac{T_{\infty }}{T_{0}}\left(\frac{dH_{T}}{dT}\right)dT}$$

In the formula, *T* corresponded to the crystallization temperature at time t; *T*_0_ and *T*_∞_ represents the initial and end temperatures of the crystallization.

In the non-isothermal crystallization process, the relationship between the crystallization temperature *T* and the crystallization time t can be calculated by formula (12).

**Figure 6:**
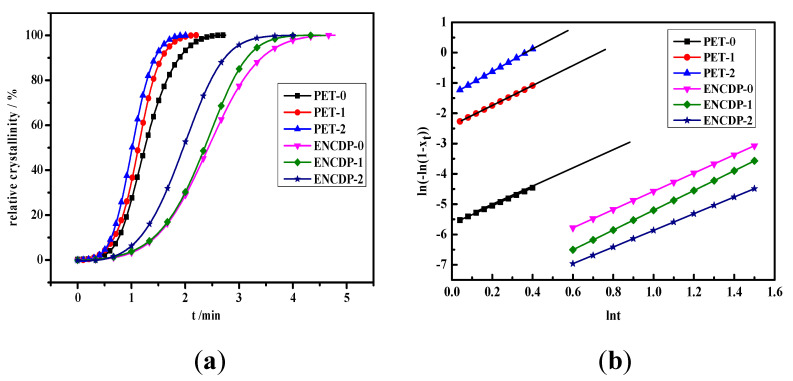
(**a**) The relative crystallinity versus time for the non-isothermal crystallization; (**b**) the *Ln*[−*Ln*(1− *X_t_*)] versus *Lnt* for the non-isothermal crystallization.

*t* = (*T*–*T*_0_)/R (12)

where R is the heating rate of the DSC program. The curves between the relative crystallinity *X_t_* and time *t* are shown in Figure 6.

Generally, the Avrami equation is one of the most common methods for investigating the isothermal crystallization behavior of the copolyester.

1− *X_t_* = exp(−*Zt*^n^) (13)

By taking the logarithm, the above equation can be calculated by formula (14).

Ln[−Ln(1− X_t_)] = LnZ + nLnt (14)

where n is the Avrami index and *Z* is the crystallization-rate constant; the plot of the *Ln*[−*Ln*(1− *X_t_*)] versus *Lnt* is shown in Figure 6b. The slope is n and the intercept of the line is *LnZ*. 

Regrettably, the Avrami equation is unsuited for describing non-isothermal crystallization process. In order to analysis the non-isothermal crystallization process, Jeziorny modied *Z*_t_ with the cooling rate, and the modified equation is as following:

(15) $$LnZ_{c}=-\frac{LnZ}{R}$$

where *LnZ*_c_ is the non-isothermal crystallization kinetic constant.

It can be seen from Figure 6a that the curves of the relative crystallinity *X_t_* versus the crystallization time *t* of all samples are in the S shape, which directly determines the semi-crystallization time (*t*_0.5_), that is, the time when the relative crystallinity reaches 50% and reflects the rate of cold-crystallization. The kinetic parameters of non-isothermal crystallization of the copolyester are deduced from Figure 6b and Equation (15), as shown in Table 8.

It can be seen from Table 8 that the *n* values are not integers, which because the homogeneous nucleation and heterogeneous nucleation may coexist during the crystallization of the copolyester, or the second crystallization may also exist. In addition, the macromolecular chain will undergo entanglement during crystallization, causing it is difficult to completely diffuse as the small molecules and the crystal growth dimension are not integers. Moreover, the Avrami exponent of the modified copolyester ENCDP-X, *n*, ranges from 2.7 to 3.2, indicating that the mode of the nucleation and growth may be two-dimensional and three-dimensional coexistence. Moreover, this shows that the addition of modified monomers SIPE, PEG and NPG did not alter the crystallization mechanism, that is, where did not exist particular nucleating agent in copolyester. The crystallization-rate constant, *Z_c_*, increases and the half crystallization time *t*_1/2_ decreases, illustrating the cold-crystallization rate increase. Moreover, the role of cold-crystallization nucleation is not significant with the increase of TiO_2_ content for copolyester ENCDP-X, due to the small difference in *Z*_c_ and *t*_1/2_. Oppositely, regarding to the copolyester (PET-0, PET-1 and PET-2), the cold-crystallization nucleation effect for TiO_2_ is significantly.

### 3.7. TG Analysis

**Figure 7:**
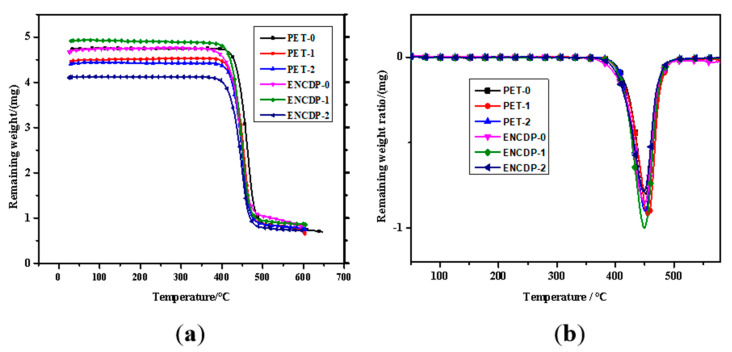
(**a**) Thermogravimetric analyzer (TGA) curve and (**b**) dTGA curve of samples.

The initial weight loss temperature (*T*_d_) at which the weight loss rate of polyester reaches 5% and the maximum thermal degradation rate temperature (*T*_dm_) are important thermal degradation parameters, which are mainly affected by the factors, such as the copolymerization component, end group contents, DEG and by-product contents in the macromolecular chain. Figure 7 shows the TGA and dTGA curves of the copolyester under the nitrogen atmosphere, and the only one thermal weightless plateau and differential peak showing the only one thermal decomposition stage during the thermal degradation of the copolyester. The corresponding thermal degradation parameters are listed in Table 9.

Relative to copolyester ENCDP-X, *T*_d_, *T*_dm_ and the thermal degradation temperature corresponding to the same thermal weight loss rate are lower, owing to the incorporation of modified monomers and TiO_2_. The modified monomers PEG and NPG not only disrupted the regularity of the macromolecular segments, but also embedded more methylene fatty segments and ether bonds. The appearance of the ether bonds enhanced the electronegativity of the oxygen atoms on the ester carbonyl groups and enhanced the positive polarity of the H atom on the methylene group at the β position easier to break the chemical bond around the ester carbonyl group and the methylene group. In addition, as the TiO_2_ proportion increases, the thermal degradation temperature corresponding to the same thermal weight loss rate will decrease to a small extent. TiO_2_-assisted thermal degradation of polymers occurs by transferring electrons from light-excited TiO_2_ to molecules to form anionic radicals. The next step involves the extinction process of free radicals, which subsequently attack the polymer. In addition, In the high temperature environment, the crystal is completely destroyed, and the molecular chain is in a free state. The TiO_2_ disrupts the regularity of polyester molecular chains, reducing the intermolecular tightness and intermolecular stress, and the corresponding chemical bonds are more easily broken [31]. 

In summary, the modified monomer is more destructive to the thermal stability of the modified copolyester. Therefore, the proportion of modified monomers should be controlled to meet spinning requirements.

### 3.8. The Contact Angle

**Figure 8:**
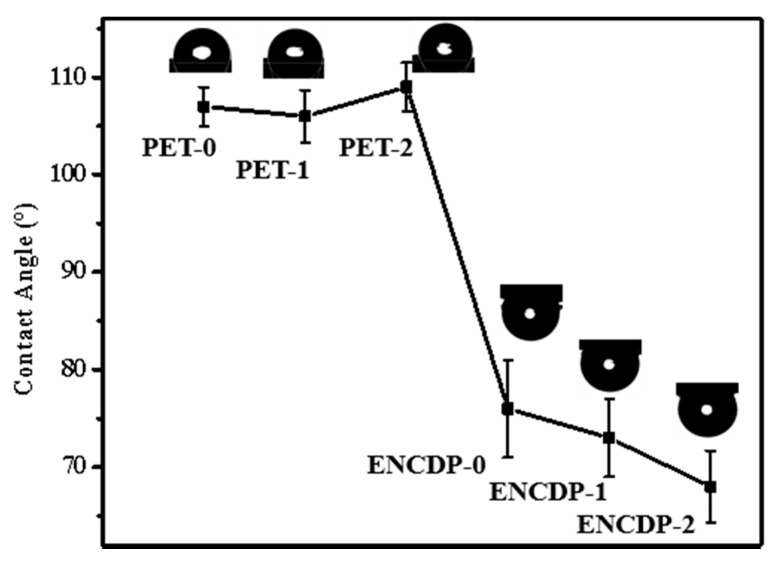
The contact angles of the copolyester.

The contact angle is an important parameter to characterize the hydrophilicity of the polymer. Its value indicates the hydrophilicity (<90°) and hydrophobicity (>90°), and the smaller the value, the better hydrophilicity. The lacking of hydrophilic groups and the closing arrangement of macromolecular chains, leading to the poor hydrophilicity for PET. The static contact angle of copolyester is shown in Figure 8.

It can be seen from Figure 8 that for the addition of the modified monomers in the copolyester ENCDP-0, the contact angle is reduced from 107° to 76°, and the polymer surface changes from hydrophobic to hydrophilic. Owing to the modified monomer is embedded in the macromolecular chain, increasing the steric hindrance effect, weakening the regularity and symmetry of the copolyester macromolecule, leading to the amorphous region and free volume being increased, increasing space for water molecules; thus, the hydrophilicity is improved for the copolyester [32]. Moreover, on the basis of copolymerization modification, TiO_2_ is further mixed to increase the passage of water molecules into the copolyester, leading to the hydrophilicity being further improved, and the contact angle being reduced from 76° to 65°. However, the blending of TiO_2_ causing the small difference for contact angle. This is because the high crystallinity and the regular macromolecular structure, causing water molecules cannot enter the crystal region. Thus, there is no obvious change in hydrophilic effect.

## 4. Conclusions

A modified poly(ethylene terephthalate) was prepared based on the incorporation of modified monomer and TiO_2_. The ^1^H-NMR spectrum indicates that most of the modified monomers (SIPE, PEG and NPG) have been successfully incorporated into the macromolecule; the monomer molar fraction is about 98%–99%, 72%–80% and 74%–82%, sequentially, not limited by TiO_2_ content. The ^13^C-NMR spectrum shows that sequential distribution and degree of randomness and analysis the resonances of quaternary carbons in the copolymers unit. The average sequence lengths of the modified monomers (SIPE, NPG) are 1.02 and 1.04, and randomness of copolyester is about 1, attributing to the random copolymerization, which is consistent with the ideal copolycondensation statistics.

The DSC curves of modified copolyester shows that the incorporation of modified monomers weakens the crystallization ability, reducing the *T*_g_, *T*_m_ and *X*_c_. The non-isothermal cold-crystallization kinetics of the copolyester by Jeziorny’s method reveals that the crystallization-rate constant, *Z_c_* increased, the half crystallization time t_1/2_ decreased, the cold-crystallization rate increased. In addition, the incorporating of TiO_2_ improves the thermal performance of the copolyester, which is beneficial to processing and application. The TGA curves indicate that the thermal degradation mechanisms are basically the same. Moreover, the addition of modified monomers and TiO_2_ reduces the thermal degradation temperature.

The copolyester exhibited a better hydrophilicity than pure PET; the contact angle was reduced from 107° to 76°. In addition, TiO_2_ was further incorporated to provide more channels for moisture entering the copolyester, leading to the hydrophilicity being further improved, and the contact angle being reduced from 76° to 65°.

## Figures and Tables

**Table 1:** Samples and its corresponding numbers.

Numbers	Samples	TiO_2_ Proportion (%)
PET-0	PET	-
PET-1	PET	0.03
PET-2	PET	0.24
ENCDP-0	ENCDP	-
ENCDP-1	ENCDP	0.03
ENCDP-2	ENCDP	0.24

**Table 2:** The intrinsic viscosity and *Mη* of the copolyesters.

Samples	[*η*] (dL∙g^−1^)	*Mη* (g∙mol^−1^)
PET-0	0.676	18,900
PET-1	0.671	18,800
PET-2	0.647	18,000
ENCDP-0	0.594	16,200
ENCDP-1	0.586	15,900
ENCDP-2	0.568	15,300

**Table 3:** The wave numbers and vibrational conformation of chemical groups.

Wave Numbers (cm^−1^)	Vibrational Conformation
3450 cm^−1^	Stretching vibration peaks for –OH
2853 cm^−1^	Stretching vibration peaks for –CH_2_– in PEG
2892 cm^−1^	feature peaks for –CH_2_–
1740 cm^−1^	The asymmetric stretching of –C=O for the aliphatic-aromatic ester units.
1578–1504 cm^−1^	Stretching vibration peaks for Benzene
1372 cm^−1^	Symmetrical deformation vibration peaks for C–H in NPG
1101,1117 cm^−1^	Absorbing vibration peaks for (C–O–C)
727 cm^−1^	Vibration peaks generated by the conjugation of benzene and –C=O
630 cm^−1^	Stretching vibration peaks for S–C in SIPE

**Table 4:** The actual reaction yield calculated from ^1^H-NMR spectra of samples.

Samples	Feed Ratio			Actual Reaction Yield		
	SIPE/PTA (mol%)	PEG/PTA (mol%)	NPG/PTA (mol%)	SIPE/PTA (mol%)	PEG/PTA (mol%)	NPG/PTA (mol%)
ENCDP-0	1.72	0.61	3.46	98.28	76.25	80.46
ENCDP-1	1.74	0.58	3.21	99.43	72.50	74.65
ENCDP-2	1.73	0.63	3.52	98.85	78.75	81.86

**Table 5:** The experimental and theoretical values for the different sequence structure.

Results	Triads Sequence (mol%)							
	BBB	BBS	SBS	BBN	NBN	SBN	SSS	NNN
Theoretical values	90.44	2.96	0.03	5.94	0.10	0.05	-	-
Experimental values	89.71	2.21	0.03	5.73	0.12	0.07	-	-

**Table 6:** Theoretical and experimental values of randomness.

Result	Av Ls		Randomness BS (%)	Av L_N_		Randomness BN (%)
	LB	LS		LB	LN	
Theoretical values	64.72	1.02	99.50	31.45	1.04	99.35
Experimental values	75.45	1.03	99.17	30.61	1.04	99.45

Label: Av Ls—Average sequence length for S (mol%), Av L_N_—Average sequence length for N (mol%).

**Table 7:** The thermal parameters of copolyesters.

Sample	*T_g_* (°C)	*T*_c_ (°C)	*T*_cc_ (°C)	*T*_m_ (°C)	△*H*_m_ (J/g)	*X*_c_ (%)
PET-0	70.17	137.69	163.18	245.59	35.82	26.38
PET-1	72.16	136.84	184.54	250.24	39.03	28.74
PET-2	72.65	134.00	196.8	251.55	40.08	29.51
ENCDP-0	64.00	154.37	-	227.94	26.70	19.66
ENCDP-1	67.67	160.19	-	234.12	27.98	20.60
ENCDP-2	67.78	156.87	148.35	234.94	28.50	20.99

Lable: the crystallinity (*X*_c_) =(Δ*H*_m_/Δ*H*_0_) × 100%, where Δ*H*_m_ is the melting enthalpy, Δ*H*_0_ denotes the melting enthalpy of PET with perfectly crystalline (Δ*H*_0_ = 135.8 J/g).

**Table 8:** The parameters of non-isothermal crystallization from Avrami analysis.

Samples	*T*_c_ (°C)	*t*_1/2_ (s)	*n*	*Z*_c_ (min^−1^)
PET-0	137.69	74.4	2.96	0.57
PET-1	136.84	67.2	3.28	0.79
PET-2	134.00	58.6	3.76	0.91
ENCDP-0	154.37	145.7	2.75	0.41
ENCDP-1	160.19	142.8	3.01	0.43
ENCDP-2	156.87	138.2	3.26	0.46

**Table 9:** Thermogravimetric data for the copolyester.

Samples	*T*_d_ (5%)	*T*_dm_
PET-0	425.48	460.89
PET-1	419.60	455.36
PET-2	417.33	450.61
ENCDP-0	411.48	449.74
ENCDP-1	410.25	448.95
ENCDP-2	410.14	448.09
